# First Chinese case report of *Helcococcus kunzii* in a patient with diabetic foot

**DOI:** 10.1186/s12879-021-06309-y

**Published:** 2021-07-20

**Authors:** Ruiying Li, Lei Xiang, Jinghui Lu, Linzhen Chen, Xiangsheng Cai

**Affiliations:** 1 Clinical Laboratory, the First Affiliated Hospital of Guangdong Pharmaceutical University, Guangzhou, China; 2 Center for Medical Experiments, University of Chinese Academy of Science-Shenzhen Hospital, Shenzhen, China

**Keywords:** *Helcococcus kunzii*, Diabetic foot, MALDI-TOF-MS, 16S rRNA gene sequencing, Case report

## Abstract

**Background:**

Infection *of Helcococcus kunzii*(*H*. *kunzii*) from diabetic foot wound is rarely reported. This case report describes the infection *of H.kunzii* and highlights the therapeutic effect on *H.kunzii* from a diabetic foot wound.

**Case presentation:**

In this study, one *H*. *kunzii* strain was isolated from a patient with diabetic foot, which was confirmed by 16S rRNA gene analysis and MALDI-TOF-MS. It is the first Chinese case of *H. kunzii* in a patient with diabetic foot. As a result of the lack of antibiotic sensitivity data and multiple comorbidities, antibiotics were used cautiously, and those administered during the first 3 months were ineffective. Then, vacuum sealing drainage (VSD) was applied during hospitalization; no antibiotics were used and the wound healed well.

**Conclusions:**

VSD alone may be more effective in treating diabetic feet infected with *H. kunzii*, which may provide reference for clinical treatment of *H. kunzii* infection from diabetic foot.

## Background

*Helcococcus kunzii* is a Gram-positive coccus that has been isolated as part of the human skin microbiota [1, 2]. However, infection of *H. kunzii* from diabetic foot wound is rarely reported. This case report describes the infection *of H.kunzii* and highlights the therapeutic effect on *H.kunzii* from a diabetic foot wound.

## Case presentation

A 66-year-old male patient was admitted to the Department of Orthopedic Surgery of our hospital on June 24, 2019 due to pain in the second toe of the left foot since January with swelling and ulceration. One month before admission, the patient felt pain in his second toe after a pedicure. The toe was red and swollen, but he ignored these signs and ulceration developed several days later. The symptoms did not improve with self-medication, and the wound increased in size. The patient’s mood was stable, his appetite and sleep were normal, there were no obvious abnormalities in stool or urine, and no significant weight loss. The patient had a 10-year history of coronary heart disease, heart failure, hypertension, and type 2 diabetes mellitus. He had experienced numbness of the limbs 2 years prior and had poor blood glucose control. Before admission, he had fever, for which he self-medicated with cefradine. His body temperature was 36.7 °C, the respiration rate was 20/min, the pulse was 89 times/min, and the blood pressure was 130/70 mmHg. The patient had good nutrition, and no facial or postural abnormalities; he cooperated fully during a physical examination. The distal phalangeal bone of the second toe of his left foot was gangrenous and grayish black, with yellowish exudation on the surface and an unpleasant odor. The skin around the wound was red, swollen, and tender; the dorsum of the foot was slightly swollen and the skin temperature was low; movement of the third toe of the left foot and the metatarsophalangeal joint was limited; the pulse of the dorsalis pedis artery was not touched and the blood supply to the distal toes was poor. An auxiliary examination at the time of admission yielded the following findings: fasting blood glucose, 7.72 mmol/L; urea nitrogen, 10.5 mmol/L; creatinine, 95 umol/L; cystatin, 2.15 mg/L, postprandial blood glucose, 9.38 mmol/L; glycosylated hemoglobin, 9.6%; white blood cell count, 9.60 × 10^9^/L, neutrophil percentage, 75.9%; C-reactive protein, 90.8 mg/L; procalcitonin, 0.171 ng/mL; IL-6, 36.47 pg/mL; and erythrocyte sedimentation rate, 127 mm/h.

**Fig. 1:**
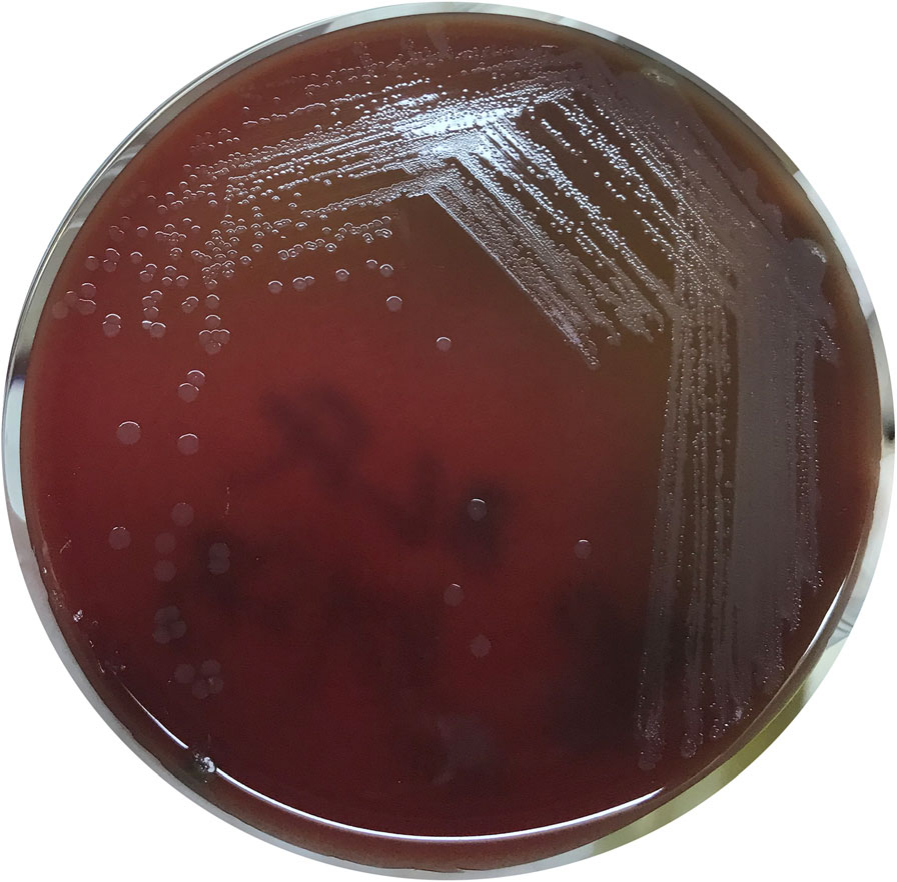
Culture colonies on blood agar plates for 72 h

**Fig. 2:**
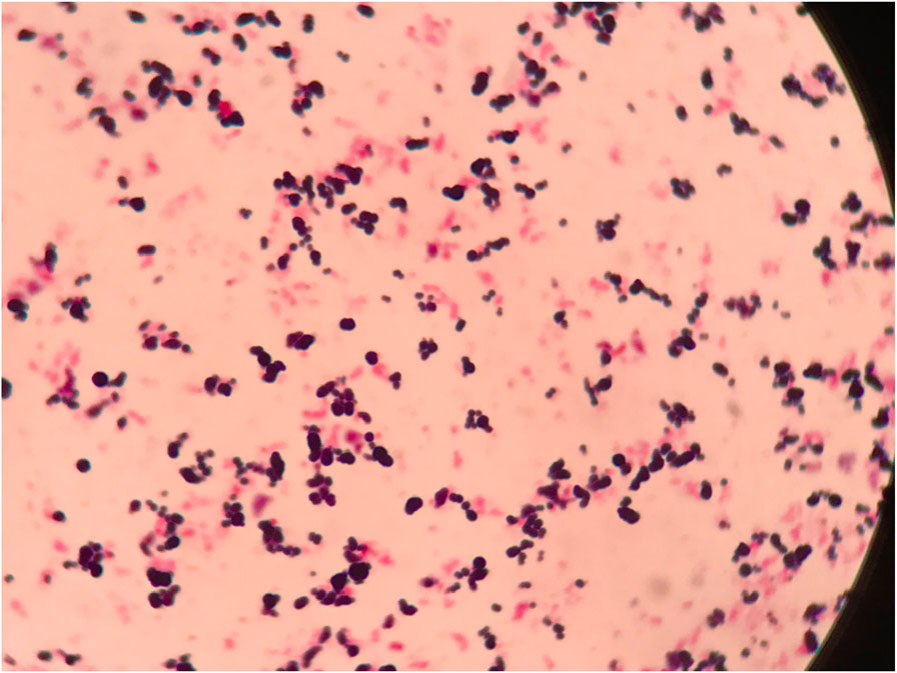
Gram stain (× 1000 times)

**Fig. 3:**
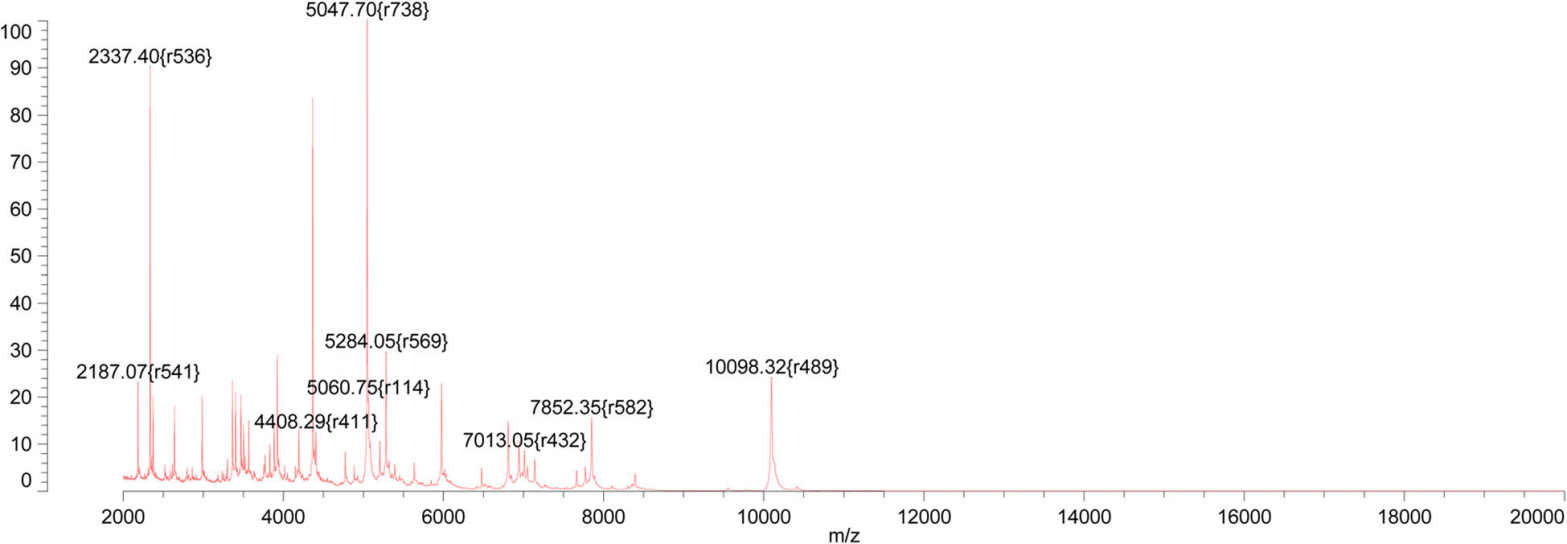
Identified as *H.kunzii* by MALDI-TOF-MS

After admission, the patient was given clindamycin, as well as agents to control his blood pressure and blood glucose level. On July 17, 2019, under spinal anesthesia, debridement of the second toe of the left foot were performed. The gangrene of the second toe was removed during the operation, and the pus cavity was found to reach the deep part of the sole. A 6-cm incision was made to remove the pus cavity fluid. After the operation, cefotiam and metronidazole were administered. During hospitalization, the secretion from the gangrene of the second toe was sampled four times for bacterial culture, and the same bacterium was isolated on July 17 and September 6, 2019. Wound-secretion samples were inoculated on Columbia blood agar and cultured at 35 °C in an atmosphere containing 5% CO_2_. After incubation for 24 h, needle tip-sized colonies could be seen. After 48 h, tiny, colorless, transparent, and dewdrop-like α-hemolytic colonies were evident. After 72 h, grayish white, translucent and slightly α-hemolytic colonies were present on the agar (Fig. 1). Large Gram-positive cocci arranged individually or in clusters were identified by Gram staining (Fig. 2). The catalase and the oxidase were negative. The isolate was transported to Guangzhou Jinyu Inspection Center for identification by matrix-assisted laser desorption ionization time-of-flight mass spectrometry (MALDI-TOF-MS); the result was *Helcococcus kunzii* (Fig. 3).

Clindamycin and cefotiam were used during hospitalization, but metronidazole was ineffective and the left-foot wound continued to deepen. In October, the patient was transferred to another hospital for further treatment. Over a 2-month period, he underwent 10 left-foot debridement and vacuum sealing drainage (VSD) procedures, and the symptoms of his underlying diseases were treated without using antibiotics. The wound surface significantly improved, necrotic tissue was virtually absent, and there was no purulent secretion.

After that, we carried out antimicrobial susceptibility testing on this bacterium. After 48 h, the diameters of the inhibition zones were as follows: ampicillin, ceftazidime, meropenem, imipenem, tetracycline, vancomycin, penicillin, cefazolin and cefoxitin, all 36 mm; and sulfamethoxazole, ciprofloxacin, amikacin, erythromycin and clindamycin, all 6 mm. Note that these data are for clinical reference purposes only because there is no drug sensitivity standard.

## Discussion and conclusions

*Helcococcus* is a facultatively anaerobic, non-motile, non-spore-forming, oxidase-negative Gram-positive coccus. It was first reported by Collins et al. in 1993 and was initially considered a component of the normal flora of human skin [1, 3, 4]. However, invasive infections by *Helcococcus* spp. have been reported in the past 20 years [5–7] . These are considered opportunistic human pathogens causing, for instance, abscess, bacteremia, cellulitis, and endocarditis, typically in immunocompromised patients with comorbidities. The *Helcococcus* species discovered to date include *H. kunzii, H. ovis, H. pyogenes, H. seattlensis*, and *H. sueciensis*. *H. kunzii* is an opportunistic pathogen of diabetic foot-wound infection. To our best knowledge, we first isolated *H.kunzii* from diabetic foot wound in our hospital and in China. Because of its slow growth, it could not be identified by the BD Phoenix system (BD, Franklin Lakes, NJ, USA); instead, it was identified as *H.kunzii* by MALDI-TOF-MS. 16S rRNA gene sequencing and BLAST alignment yielded coincidence rates of our isolate with KM403387.1, KR185383.1, and MG188744.1 *H. kunzii* of 98, 97 and 93%, respectively.

Because human infection by *H. kunzii* is rare, its antibiotic sensitivity profile is unclear. Park et al. [8] reported that *H. kunzii* was sensitive to penicillin, ampicillin, ampicillin/sulbactam, ertapenem, meropenem, and piperacillin on Etest, but resistant to clindamycin, erythromycin, and metronidazole. Using Kirby-Bauer (KB) disk diffusion assay, Jorge et al. [9] found that a *H. kunzii* strain was sensitive to ampicillin, cefazolin, clindamycin, ciprofloxacin, vancomycin, trimethoprim, sulfamethoxazole, rifampicin, and linezolid, and resistant to erythromycin and gentamicin. In this study, after 48 h, the diameters of the inhibition zones were as follows: ampicillin, ceftazidime, meropenem, imipenem, tetracycline, vancomycin, penicillin, cefazolin and cefoxitin, all 36 mm; and sulfamethoxazole, ciprofloxacin, amikacin, erythromycin, and clindamycin, all 6 mm. Note that these data are for clinical reference purposes only because there is no drug sensitivity standard.

In conclusion, we report the first Chinese case of *H. kunzii* confirmed by 16S rRNA gene analysis and MALDI-TOF-MS. As a result of the lack of antibiotic sensitivity data and multiple comorbidities, antibiotics were used cautiously, and those administered during the first 3 months were ineffective. Then, VSD was applied during hospitalization; no antibiotics were used and the wound healed well*.* Therefore, VSD alone may be more effective in treating diabetic feet infected with *H. kunzii*, which may provide reference for clinical treatment of *H. kunzii* infection from diabetic foot.

## Data Availability

The raw data supporting the conclusions of this article will be made available by the authors, without undue reservation**.**

## Abbreviations

VSD: Vacuum sealing drainage
*H.kunzii*: *Helcococcus kunzii*
C: Degrees Celsius
L: Liter
mL: Milliliter
mg: Milligram
ng: Nanogram
pg: Picogram
h: Hour
mmol: Millimole
umol: Micromole

## References

[1] Haas J, Jernick SL, Scardina RJ, Teruya J, Caliendo AM, Ruoff KL (1997). Colonization of skin by Helcococcus kunzii. J Clin Microbiol.

[2] Lemaître N, Huvent D, Loïez C, Wallet F, Courcol RJ (2008). Isolation of Helcococcus kunzii from plantar phlegmon in a vascular patient. J Med Microbiol.

[3] Collins MD, Facklam RR, Rodrigues UM, Ruoff KL (1993). Phylogenetic analysis of some Aerococcus-like organisms from clinical sources: description of Helcococcus kunzii gen. Nov., sp. nov. Int J Syst Bacteriol.

[4] Caliendo AM, Jordan CD, Ruoff KL (1995). Helcococcus, a new genus of catalase-negative, gram-positive cocci isolated from clinical specimens. J Clin Microbiol.

[5] Collins MD, Falsen E, Brownlee K, Lawson PA (2004). Helcococcus sueciensis sp. nov., isolated from a human wound. Int J Syst Evol Microbiol.

[6] Collins MD, Falsen E, Foster G, Monasterio LR, Dominguez L, Fernandez-Garazabal JF (1999). Helcococcus ovis sp. nov., a gram-positive organism from sheep. Int J Syst Bacteriol.

[7] Chow SK, Clarridge JE (2014). Identification and clinical significance of Helcococcus species, with description of Helcococcus seattlensis sp. nov. from a patient with urosepsis. J Clin Microbiol.

[8] Park JH, Woo BM, Hong SK, Kim EC (2014). First Korean case of Helcococcus kunzii bacteremia in a patient with diabetes. Ann Lab Med.

[9] Pérez-Jorge C, Cordero J, Marin M, Esteban J (2012). Prosthetic joint infection caused by Helcococcus kunzii. J Clin Microbiol.

